# Missionaries, measles, and manuscripts: revisiting the Whitman tragedy

**DOI:** 10.5195/jmla.2019.538

**Published:** 2019-01-01

**Authors:** Melanie J. Norton, John Booss

**Affiliations:** Head of Access and Delivery Services, Cushing/Whitney Medical Library, Yale University School of Medicine, New Haven CT, melanie.norton@yale.edu; Departments of Neurology and Laboratory Medicine, Yale University School of Medicine, New Haven, CT, john.booss@yale.edu Professor Emeritus

## Abstract

The missionaries Marcus Whitman, a doctor, and Narcissa Whitman, his wife, and twelve other members of the Waiilatpu Mission were murdered in November 1847 by a small contingent of the Cayuse Indians in the Oregon Territory. The murders became known as the “Whitman Massacre.” The authors examine the historical record, including archived correspondence held at the Yale University Libraries and elsewhere, for evidence of what motivated the killings and demonstrate that there were two valid perspectives, Cayuse and white. Hence, the event is better termed the “Whitman Tragedy.” A crucial component, a highly lethal measles epidemic, has been called the spark that lit the fuse of the tragedy.

## INTRODUCTION

This article is derived from a fortunate instance of serendipity. On investigating what had been described as the “Whitman Massacre” and the role of measles in provoking the killings, author Booss noted that a book he had been studying needed repair and restoration. On his returning the book to Yale University’s Cushing/Whitney Medical Library, author Norton observed that she might have a family connection to certain victims of that tragedy: the Sager children. The following project developed from the discussions that ensued. Fortunately, there is a wealth of manuscripts, held at the Beinecke Library at Yale and elsewhere, from which to evaluate the tragedy. Leaving the issue of the Sager children for a later investigation, we instead examined the issues and beliefs that separated the Natives and the American missionaries. It emerged that each issue had two perspectives, two truths. We concluded that the event is better described as the “Whitman Tragedy.” We tell that story and describe the two truths in this article.

## RESEARCH AND DISCUSSION

On the morning of November 29, 1847, a group of Cayuse Indians gathered at a Calvinist Protestant Mission, Waiilatpu, in the Oregon Territory. The mission had been established by Dr. Marcus Whitman and Narcissa Whitman, his wife, eleven years earlier. They felt strongly motivated to save Indian souls with Christianity. Of late, there had been strong discontent among the Indians. The missionaries themselves were turning their efforts toward supporting American emigrants streaming west over the Oregon Trail.

The mission was a place with which the Indians were familiar and at which they were recognized. Hence, their arrival on that morning caused little stir. The doctor was exhausted, having returned the previous evening from attending to Indians who were in the throes of a deadly measles epidemic. That measles epidemic was the proximate cause of the actions that followed. An Indian known as Tomahas aided by another, Tiloukait, buried a hatchet in the skull of the unsuspecting Marcus. Narcissa, too, would be murdered on that deadly November morning along with twelve other members of the mission community [1].

Women and children were taken prisoner, to be ransomed later by Peter Skene Ogden of the Hudson’s Bay Company. It was a premeditated, vicious attack that originally entered the annals of the Pacific Northwest as the “Whitman Massacre.” However, two perspectives must be considered, and the event is better termed the “Whitman Tragedy.” Among the many factors, the Indians’ views on the measles epidemic were crucial.

The events at the Whitman Mission encapsulated the relentless drive of Euro-Americans west to the Pacific Ocean, known as “manifest destiny.” Originally, the conquest of the American continent, the “new world,” was justified by a Christian doctrine of discovery and conquest. In the drive west, lands were wrested from their original indigenous inhabitants. With them came the introduction of European infectious diseases to indigenous peoples, as if to a virgin field. Witnessing the ever-increasing stream of eastern immigrants and the diseases they brought, the Cayuse feared the ultimate loss of population and of their own lands.

With the benefit of primary sources, we examined the Indians’ fear and the other components of the conflict with the Whitmans. We found that the personalities of the Whitmans and the strict unbending belief system of Calvinist religion in which they were embedded played major roles in the conflict of perspectives. The collections of primary documents allowed the two perspectives to emerge. Without the collections of primary documents, we would be dependent on secondary sources and the bias of the times in which they were compiled.

In this age of cell phones, texting, Skype, and a twenty-four-hour news cycle, one strains to comprehend the importance of letters, which sometimes took months to reach their destinations, in order to remain connected with families and friends a continent away. Both Marcus and Narcissa were well educated, loyal correspondents and anxious to report their progress to friends and family in the east. Much of that correspondence and that of others relating to them has been preserved as manuscripts in archives at the Oregon Historical Society, Whitman College, and the Coe Collection at the Beinecke Library at Yale University. While explicitly preserving the Whitmans’ voices, much of the correspondence reports on the discontent of the Indians, indirectly giving their perspective. We relied on that correspondence and on other historical sources. More recently, the two perspectives can be found in two museums: the Native American based Tamastslikt Cultural Institute and the Whitman National Historic site.

The many factors leading up to the tragedy were interlocking, making it seem almost inevitable. Land was a key issue with Indians, with whites having very different concepts about it. Closely linked to differing concepts about the land was the ever-increasing size of the immigrant wagon trains going through and despoiling Cayuse territory. The Cayuse feared that ultimately their lands for hunting and gathering would be lost to the whites.

The Indian views conflicted with Euro-American concepts of property ownership. The connection to land for Indians was spiritual but was viewed as pagan by white people. The Christian religion was also at odds with Indian views of spirituality and health. Shamans or te-wats, Indian medicine men, were believed not only to have the power to remove the spiritual causes of disease, but also to induce evil spirits through sorcery. In that situation and in the event of a te-wat failing to cure a sick relative, the price on rare occasion would be death for the medicine man. The notion of evil spirits, believed to be under Marcus’s control, played a major role in the Cayuse response to the measles epidemic. The disease carried away about 40% of the tribe, many more than the whites. It was an example of an infection sweeping through an unprotected population, later termed a “virgin soil epidemic” [2]. That disparity would put him at risk as if he were a te-wat.

With respect to appropriation of the land, Euro-Americans, from the first trappers to the influx of settlers, behaved as if the land was theirs for the taking, disregarding the rights of the indigenous peoples who had lived on the land for eons. At least two theoretical constructs underlay this outrageous racist activity. A Papal Bull issued in 1493 articulated the Doctrine of Discovery, which laid claim to lands that were not under Christian control. More explicitly, New England Puritans articulated a doctrine of *“vacuum domicilium,”* the right to take vacant land to make it productive and make it prosper. Thus, Indian lands on which they hunted, fished, trapped, and gathered were seen by Euro-Americans as vacant and undeveloped. The fencing-off of property, the reduction of game, and the destruction of camas roots, a vital Indian food source, by domesticated pigs that were allowed to roam were all considered harbingers of the death of their way of life for the Cayuse.

While the goal of most of the increasing flood of immigrants passing on the Oregon Trail in the 1840s was the Willamette Valley further west, the Cayuse worried that their own lands and way of life were in danger. In a letter of November 25, 1845, held by the Coe Collection at the Beinecke Library, Marcus Whitman wrote to a missionary colleague of an Indian’s grave concerns [3]. The historian, C.M. Drury, stated that Whitman gave “the Indians’ side of the story.” The Indian, Young Chief, “spoke of the Americans as having a design to obtain their country & property.” Young Chief also complained of new diseases that the white man had introduced to the Indians. As Drury put it, “the white men were prepared ‘with poison and infection’ to kill off the Indians in order to gain possession of their lands and horses” [1].

In 1842, the sponsoring organization of the Whitman and related missions, the American Board for Foreign Missions based in Boston, decided to close the mission at Waiilatpu and reassign the personnel to the other missions. Marcus undertook a remarkable journey east in the fall and winter of 1842/43 to challenge the decision, while Narcissa was to stay at the mission. Marcus was successful in arguing that the mission should remain open and then journeyed to Washington, DC, to plead the case for American emigration to Oregon. While the extent of his influence on the Oregon question has been disputed, it certainly emphasized the importance of Oregon to the national agenda. On his return trip to Oregon, he joined and ultimately led the emigration wagon train of 1843. It was estimated to have up to 1,000 emigrants and 120 wagons. Subsequent wagon trains in the 1840s were of ever-increasing size. From that time, the Whitmans appeared to shift much of their attention to supporting the wagon trains and less to saving the souls of the Indians. Young Chief’s concerns were well founded.

While they were passionate about their professed Christian calling and devoted their lives to it, Marcus and Narcissa failed. The Cayuse were not converted. This disappointing result must be understood in the context of the impediments faced. Two of the most formidable were the native way of life and their languages. Life for the Indians was semi-migratory and tied to what has been called “the seasonal round.” Thus at various times, Indian men would go off to hunt elk, antelope, and deer. At other times, while the massive herds of buffalo still existed on the Great Plains, the Indian men would go over the mountains to hunt. Trips to tributaries of the Columbia River at the time of salmon runs were a part of life for some Cayuse. At appropriate seasons, Cayuse women would collect berries and dig for the tubers of camas plants. The Whitmans and other missionaries struggled against the Cayuse form of life and attempted to get them to establish farms and adopt a settled agricultural life. The missionaries seemed to believe that a fixed location would promote the adoption of Christianity. Both of the Whitmans hailed from a farming region in western New York State. The futility of the “civilize and Christianize” task, a residue of the settling of New England, is apparent.

Coupled with the semi-migratory way of life of the Cayuse was the difficulty of transmitting the complex concepts of the Christian faith into Cayuse and the related language spoken by the Nez Perce. While Marcus achieved some facility in the language of the Nez Perce, Narcissa did not. Some concepts—such as original sin, atonement, and the holy trinity—did not exist in native speech. In addition, the unforgiving nature of Calvinist piety must have been repugnant to the Cayuse. The direction to give up polygamy was a persistent source of disagreement. In contrast to a system of sin and the afterlife of Christianity, the Indians believed in spirits, from those permeating the environment to personal spirits. Shamans interpreted the meanings of omens and dreams to predict the future and to determine when important activities should begin. When those conflicts in belief systems are coupled with the progressive erosion of their way of life as immigrant wagon trains poured through their hunting grounds, there is little wonder that the Whitmans’ efforts to Christianize the Cayuse failed. Not only did their efforts fail, but in the process put the Whitmans in jeopardy.

Probably nowhere were the contrasts in belief systems greater than in the understanding of diseases, especially epidemic diseases, and in the means of treating them. In the first half of the nineteenth century, Europe was on the cusp of the germ theory of Louis Pasteur in France and Robert Koch in Germany. Based in Hippocratic thought, most of Euro-American medicine until then was oriented toward material concepts. Treatments included bleedings, purgatives, and various other interventions to balance the humors. For Native peoples of the Columbia Plateau, illness was attributed to evil spirits that had invaded the body or to the loss of one’s own personal spirit. It was the task of the shaman to extirpate evil spirits.

Shamans held highly privileged positions in Indian society. Recognition of their special powers came early in life after successful spirit quests in which they had encountered their personal spirit and later when their special powers to manipulate spirits became apparent. They attempted removal of evil spirits through healing ceremonies, prayer, rituals, and incantations. Their interventions included herbal remedies and sweat lodges. It was believed that use of the sweat lodge followed by immersion in cold streams hastened death from severe illness and contributed in part to the markedly greater mortality of Indians during epidemics.

While shamans held privileged positions, that privilege also carried some risk. Natives believed that shamans with sufficient power could cause disease as well as cure it. Under certain circumstances, such as when they failed to cure and death resulted, they were suspected of killing. The incursion of European infectious illnesses proved particularly challenging for shamans. Epidemics of smallpox and measles with very high rates of mortality carried away whole villages and could reduce native populations to mere shadows of their former selves. Since shamans were believed to hold the power to kill, they were at risk of being killed in revenge. The Whitmans were aware of this practice as shown in a letter held by the Oregon Historical Society in Portland, Oregon. On May 3, 1837, a decade before the Whitman murders, Narcissa wrote to her family of the killing of a te-wat after unsuccessful treatment of an old war chief, whose death was thus considered avenged.

Measles and dysentery struck the Columbia Plateau as part of the epidemic in the Pacific Northwest in 1847–1848 [4]. Modern studies tracked measles north from California. Previously, it had been documented on wagon trains. The Cayuse were thought to have brought it to the Whitman Mission. It has been estimated that 40% of the Cayuse died, while the numbers at the mission were much lower. Natives believed that Marcus bore some responsibility for the deaths, in the same manner as would a te-wat. Furthermore, some Indians had been accidentally poisoned previously, adding to the suspicion. In point of fact, Marcus had been worn out attempting to help the Indians deal with the epidemic as well as the people at his own mission. The Cayuse drew the opposite interpretation, and Marcus was murdered with his wife and twelve others near the height of the epidemic. Historian Drury judged that the measles epidemic was the major cause of the killings. It has been said by RC Heizer that measles was the spark that lit the fuse.

One of the key questions was the role of the Whitmans themselves. What effect did their personalities and their actions play leading up to the Whitman Tragedy? Narcissa Prentice and Marcus Whitman each felt called to serve the Lord. Both were from western New York State, a farming district known as the “Burned Over District.” It had earned that name because of a series of religious revivals in the first third of the nineteenth century. The time in which the Whitmans grew up was permeated by the religious fervor of the Second Great Awakening.

Narcissa Prentice felt the call to save the heathens. On February 23, 1835, she “offered herself” to the American Board of Commissioners for Foreign Missions: “Having found favor of the Lord and desiring to live for the conversion of the world, I now offer myself to the American Board to be employed in their service among the heathen, if counted worthy” [5]. Marcus was also driven to save the souls of pagans. He had wanted to train for the ministry but was denied the opportunity by his uncle who urged him to become a medical doctor. He trained for medicine at the Fairfield Medical College, officially known as the College of Physicians and Surgeons of the Western District of New York. The medical college was incorporated in 1812 and closed in 1841. Whitman developed good experience as a doctor and was counted a fine physician. Turning toward his original passion, he applied to the American Board of Commissioners for Foreign Missions, but his first application was rejected because of putative ill health and the lack of a need for a physician. It was later approved as his health had improved.

A common interest in saving souls and the proximity of their towns in New York State afforded the opportunity for Narcissa and Marcus to meet. They formed a friendship and ultimately became engaged and married. Since the Whitmans had grown up in an era of religious fervor and had come from a region known for its revivalist enthusiasms, there was little wonder that they chose to devote their lives to saving souls among the “heathen.” To give up their middle class lifestyle and head into unknown territory was courageous. It is difficult at this remove in time to understand the fervent religious conviction that would drive such a decision. It is even more difficult to understand how things had ended up so badly after such a committed beginning.

After the murders of the Whitmans, Reverend HKW Perkins attempted to explain to Narcissa’s sister, Jane, the role of the Whitman personalities in the tragedy. Perkins had been a Methodist minister in Oregon at The Dalles. Narcissa had been invited to live with him and his wife during the winter that Marcus went east to convince the American Board to change its decision about Waiilatpu. Hence, he had had a good opportunity to learn about the Whitmans’ characteristics and to evaluate their roles in the subsequent tragedy. Drury came across a copy of the letter in the archives of Whitman College. Perkins wrote:

Both herself [Narcissa] & her husband were out of their proper sphere. They were not adapted to their work. They could not possibly interest & gain the affections of the Natives…though they *feared* the Doctor they did not love him…

He looked upon them as an inferior race & doomed at no distant day to give place to a settlement of enterprising Americans…Indeed it might almost be doubted whether he felt half the interest in the Natives that he did in the *prospective* white population.

Perkins was just as severe in assessing Narcissa:

Mrs. Whitman was not adapted to savage but to *civilized* life…The natives esteemed her as proud, haughty, as *far above them*…She longed for society, *refined society*…She was not a *missionary*, but a *woman*, an American highly gifted, polished American lady. And such she died. [italics as published]

## SUMMARY

**Figure 1:**
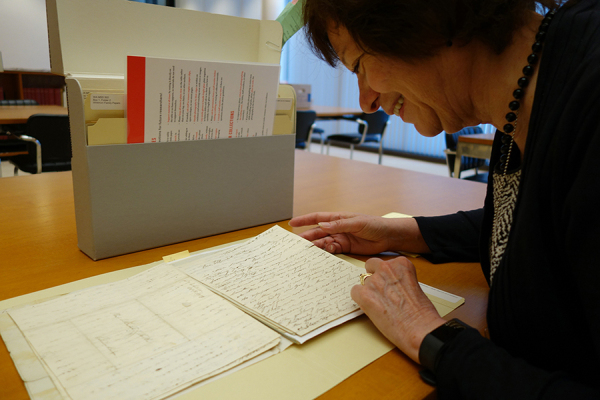

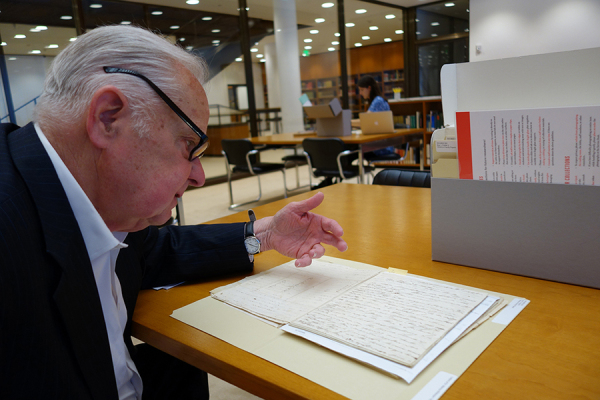
Authors Melanie J. Norton (left) and John Booss (right) examining Whitman Correspondence at the Beinecke Library

We have examined the several factors contributing to the murders of the missionaries in the Whitman Tragedy, which was triggered by a measles epidemic. Distinct perspectives were found. Correspondence held in major archives including that of the Coe Collection at the Beinecke Library at Yale University, the Oregon Historical Association, and the Whitman College Archives offered insights into the Whitmans’ aspirations and fears. It also demonstrated the perspectives of the Cayuse Indians, including fears about the introduction of diseases. Examination of the original correspondence at the Coe Collection—the paper, ink and hand writing, in addition to the narrative content—lent a sense that one was in the presence of history itself (Figure 1). The creation and permanent retention of such archives of primary documents allows scholars to return with original questions as historical studies advance.
